# Accuracy of height and weight estimation by critical care staff

**DOI:** 10.1186/cc11130

**Published:** 2012-03-20

**Authors:** K Dunne, S Hickey

**Affiliations:** 1 Forth Valley Royal Hospital, Larbert, UK

## Introduction

Patient's height and weight measurements are used regularly within the critical care setting whether for calculation of drug doses, nutritional intake, ventilator settings or calibration of cardiac output monitoring [1]. In sedated patients these parameters are often obtained via estimation by critical care staff. Errors in these estimations have the potential to cause harm either from errors in drug calculations [2], inappropriate ventilatory settings or underfeeding or overfeeding.

## Methods

We asked members of the critical care team (medical, nursing staff, physiotherapists and dieticians) to anonymously estimate the heights and weights of patients within the unit at that time. Following this we obtained accurate measurements by measuring height with a measuring tape and patients' weight with the Scotweigh weighing machine. The results were then collated and the percentage inaccuracy of estimate compared to actual measurement was calculated.

## Results

**Figure 1:**
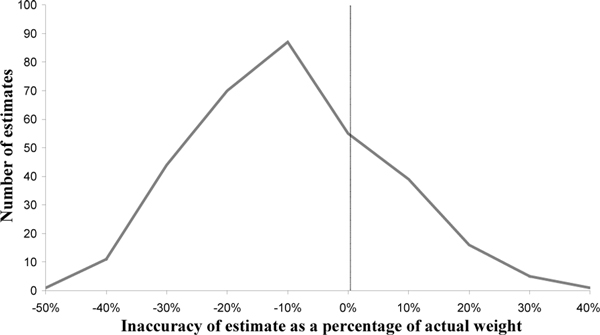
**Accuracy of weight estimation by critical care staff**.

There were 330 estimations made by 30 members of staff. Height estimation was accurate ±10% for 291 patients (88.4%). Inaccuracy in height estimation ranged from -9.5% to +25% with a mean inaccuracy of 4.75%. Weight estimation was accurate ±10% for 123 patients (38.4%). Inaccuracy of weight estimation ranged from -48.9% to +40.3% with a mean inaccuracy of 16.4%. There was a tendency to underestimate weight with only 33 estimates (10%) greater than 10% of actual weight and 174 estimates (52.7%) less than 10% of actual weight. See Figure 1.

## Conclusion

Although height estimation was measured to within 10% accuracy in the majority of cases, staff were considerably less reliable at estimating an accurate patient weight and on more than one-half of all estimates underestimated the weight by greater than 10%. We therefore strongly discourage the practice of weight estimation in situations where clinical decisions are being based on an often unreliable value, and alternative means of obtaining an accurate weight measurement should be sought.
